# Comparative study of the effect of neuromuscular electrical stimulation and oral administration of branched-chain amino acid on preventing sarcopenia in patients after living-donor liver transplantation: study protocol for an open-label randomized controlled trial

**DOI:** 10.1186/s13063-021-05086-y

**Published:** 2021-02-12

**Authors:** Masafumi Haraguchi, Kunihiro Ichinose, Hisamitsu Miyaaki, Masatoshi Hanada, Masanori Fukushima, Ryu Sasaki, Satoshi Miuma, Takanobu Hara, Tota Kugiyama, Akihiko Soyama, Masaaki Hidaka, Ayumi Tsuji, Rintaro Yano, Motohiro Sekino, Hideaki Takahata, Susumu Eguchi, Kazuhiko Nakao

**Affiliations:** 1 Department of Gastroenterology and Hepatology, Nagasaki University Graduate School of Biomedical Sciences, 1-7-1 Sakamoto, Nagasaki City, Nagasaki 852-8501 Japan; 2 Department of Immunology and Rheumatology, Division of Advanced Preventive Medical Sciences, Nagasaki University Graduate School of Biomedical Sciences, Nagasaki City, Nagasaki Japan; 3 Cardiorespiratory Division, Department of Rehabilitation Medicine, Nagasaki University Hospital, Nagasaki City, Nagasaki 852-8501 Japan; 4 Department of Surgery, Nagasaki University Graduate School of Biomedical Sciences, Nagasaki City, Nagasaki 852-8501 Japan; 5 Department of Nurse, Nagasaki University Hospital, Nagasaki City, Nagasaki 852-8501 Japan; 6 Division of Intensive Care, Nagasaki University Hospital, Nagasaki City, Nagasaki 852-8501 Japan

**Keywords:** Branched-chain amino acids, Decompensated cirrhosis, Living donor liver transplantation, Neuromuscular electrical stimulation, Sarcopenia

## Abstract

**Background:**

Liver cirrhosis is the irreversible fibrosis of the liver and causes refractory ascites and hepatic encephalopathy, which might not respond to treatment. Living donor liver transplantation (LDLT) is an effective treatment for patients with cirrhosis. However, post-LDLT patients are prone to muscle atrophy and sarcopenia. Therefore, physiotherapy of post-LDLT patients is essential for preventing the progression of sarcopenia. Recently, rehabilitation using neuromuscular electrical stimulation (NMES) has been reported to be useful for preventing the progression of sarcopenia. Similarly, nutrition therapy is essential for post-LDLT patients because these patients frequently experience malnutrition. However, the effects of combined NMES and nutrition therapy on post-LDLT patients remain unknown.

**Methods/design:**

This open-label, randomized, parallel-group study will compare the effects of combined therapy with NMES and branched-chain amino acids (BCAA) with those of NMES alone in patients with decompensated cirrhosis after LDLT. After LDLT, 50 patients with decompensated cirrhosis will be randomly assigned to receive NMES with BCAA or NMES without BCAA. The duration of the intervention will be 3 months. To analyze the change in skeletal muscle mass, InBody 770 body composition and body water analysis and ultrasonography will be performed before LDLT and 4 weeks and 12 weeks post-LDLT. The primary endpoint is changes in the skeletal muscle mass from baseline to 3 months. Important secondary endpoints are the changes in the skeletal muscle mass from baseline to 1 month and changes in the quadriceps strength from baseline to 1 month.

**Discussion:**

The results of this study are expected to provide evidence regarding the effect of NMES combined with BCAA therapy on the skeletal muscle of post-LDLT patients.

**Trial registration:**

Japan Registry of Clinical Research jRCTs071190051. Registered on February 26, 2020.

## Background

Liver cirrhosis is the irreversible fibrosis of the liver; it has various causes and is classified into compensated cirrhosis and decompensated cirrhosis [1]. Patients with decompensated cirrhosis often experience complicating refractory ascites and hepatic encephalopathy, which might not respond to systemic treatment [2, 3]. Living donor liver transplantation (LDLT) is an effective treatment for patients with decompensated cirrhosis. However, such patients often develop muscle atrophy and sarcopenia, leading to decreased quality of life [4–6]. Exacerbation of sarcopenia affects the prognosis of patients with cirrhosis [7]. Moreover, after LDLT, there are many factors that limit post-operative weaning, such as an unstable fluid balance due to decreased hepatic metabolism and associated retention of pleural effusion and ascites [8], which lead to muscle atrophy and sarcopenia.

Physical therapy is considered essential post-LDLT to facilitate motor function recovery [9–11]. Additionally, physical performance potentially increases with the recovery of liver function [12]. Since recovery after transplantation differs markedly across patients, no specific protocols have been established for postoperative physical therapy.

Recently, rehabilitation using neuromuscular electrical stimulation (NMES) has been reported to be useful for preventing the decline in the activities of daily living (ADLs) [13]. NMES is a practical, passive therapy that can be safely applied to maintain quadriceps muscle mass in seriously ill patients [14]. We have previously reported that NMES, combined with conventional physical therapy, significantly improved muscle mass in post-LDLT patients, compared with physical therapy alone [15].

Furthermore, after LDLT, patients are frequently malnourished. Hence, nutrition therapy, as well as rehabilitation, is essential after LDLT. A previous report indicated that nutritional status and metabolism were improved by adding branched-chain amino acids (BCAA) for 4 weeks after LDLT [16]. However, the effects of a combination of BCAA and NMES on patients after LDLT remain unknown. Therefore, there is a need to evaluate the effects of postoperative adjuvant therapies, such as physical therapy and nutritional therapy, on preventing the decline in ADLs due to muscle atrophy or exacerbation of sarcopenia after LDLT in patients with decompensated cirrhosis.

This study will aim to determine whether NMES and BCAA combination therapy can contribute to the recovery of skeletal muscle mass in patients with decompensated cirrhosis after LDLT.

## Methods/design

### Study design

**Fig. 1:**
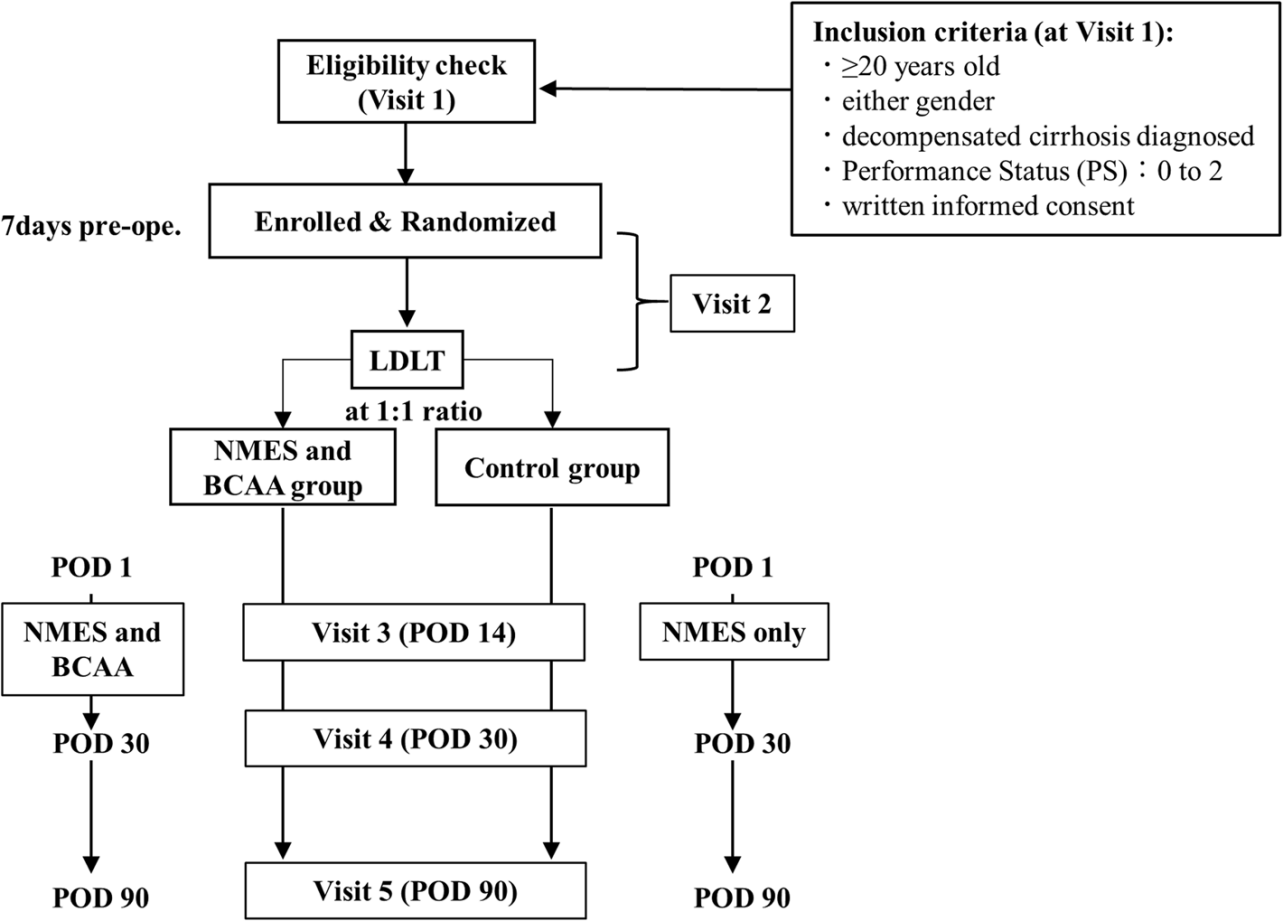
Study design. An eligibility check is conducted, and the patient’s informed consent is obtained at visit 1. The patients who satisfy the inclusion criteria are enrolled in the study at visit 2; they are randomized in a 1:1 ratio to the NMES and BCAA group (administered Aminoleban EN 100 mg) and the control group. BCAA, branched-chain amino acids; LDLT, living donor liver transplantation; NMES, .neuromuscular electrical stimulation

This is a pilot trial to assess the effect of NMES and BCAA combination therapy on skeletal muscle mass. We have designed a single-center, randomized, open-label, parallel-group, active-controlled trial for patients with decompensated cirrhosis scheduled to undergo LDLT. We will randomly assign 50 patients, in a 1:1 ratio, to the NMES and BCAA group or the control group (receiving treatment with NMES only). The overall objective is to test for the superiority of the NMES and BCAA group compared with the control group. The study follow-up duration will be 3 months. The study will be conducted at Nagasaki University Hospital, Japan, and the study design is summarized in Fig. 1.

### Outcomes

The primary outcome measures are changes in the skeletal muscle mass, as determined using the InBody 770 (InBody, Tokyo, Japan). For this parameter, any change will be determined based on the difference between the measurement results at baseline (day 0) and 3 months post-LDLT.

The study’s secondary outcome measures are as follows: changes in the skeletal muscle mass from baseline to 1 month post-LDLT; changes in the Skeletal Muscle Index (SMI), as determined by the InBody 770; changes in quadriceps muscle thickness, as determined by real-time B-mode ultrasonography (ProSound 2; Hitachi-Aloka Medical Co., Tokyo, Japan); quadriceps strength, as determined by a handheld dynamometer fixed with a belt (μ-Tas F^−1^; Anima Corporation, Tokyo, Japan); and handgrip strength from baseline (day 0) to 1 month and 3 months post-LDLT.

### Sample size estimation

This is a trial to assess the changes in skeletal muscle mass by NMES and BCAA combination therapy as compared with NMES only. There is no prior similar study that can be used to estimate the precise optimal sample size. We estimated sample size with reference to a study published by Koya et al. in 2017 [17]. This was a retrospective study comparing the skeletal muscle mass before and after transarterial chemoembolization (TACE) in 54 hepatocellular carcinoma patients who were rehabilitated for 1 week after TACE; the study reported that the presence or absence of BCAA affected changes in muscle mass. We set the number of cases based on the average and the variance-covariance matrix of the SMM value, before and after treatment in the BCAA group and the non-BCAA group, obtained from this study. The justification for this sample size is based on the rationale concerning feasibility and precision regarding the mean and variance of the primary outcome measures.

### Patients and public involvement statement

There is no patient or public involvement in this trial.

### Participants and recruitment

Fifty participants aged ≥ 20 years will be recruited into the study. The enrollment started in February 2020. All participants have been diagnosed with decompensated liver cirrhosis and will undergo LDLT. Patients who fulfill the inclusion criteria described below will be invited for eligibility screening. The transplant coordinator informs investigators of the patients scheduled for LDLT. The principal investigator and co-investigators recruit the participants among these patients and obtain written informed consent from the participants. The principal investigator is responsible for obtaining informed consent, and there are no additional consent provisions for the collection and use of participant data and biological specimens in ancillary studies. The recruitment rate and the consent rate will be evaluated at the end of the study.

### Inclusion criteria

As shown in Fig. 1, participants must fulfill the following criteria to be eligible for inclusion at their first visit to Nagasaki University Hospital (visit 1): (*1*) aged ≥ 20 years, (*2*) either sex, (*3*) diagnosis of decompensated liver cirrhosis, (*4*) Performance Status (ECOG PS) of 0–2, and (*5*) written informed consent provided.

### Exclusion criteria

Eligible participants will be excluded if they meet any of the following criteria: patients with decompensated liver cirrhosis (*1*) complicated by a bone or a joint disorder that might affect the effectiveness of NMES; (*2*) complicated by a fragile skin disease, which might suffer damage due to NMES; (*3*) complicated with a cerebrovascular disorder; (*4*) undergoing hemodialysis; (*5*) complicated by amino acid metabolism abnormalities; (*6*) who are pregnant or lactating; (*7*) participating in other clinical research within 4 months before enrollment; (*8*) complicated by chronic heart disease or using a pacemaker; (*9*) complicated by malignancy other than hepatocellular carcinoma; (*10*) complicated by active infections or tuberculosis; (*11*) complicated by severe hypertension; (*12*) complicated by extreme weakness; (*13*) who will undergo Deceased Donor Living Transplantation; (*14*) who are being included in other trials (all drugs and interventions are permitted during this trial); and (*15*) otherwise deemed inappropriate by the study’s principal investigator.

### Withdrawal criteria

Participants will be withdrawn from this trial after randomization if they meet any of the following criteria: (*1*) if they have requested a withdrawal from the study; (*2*) if it is determined by the investigators that it is inappropriate for the patient to continue participating in the study because of the progression of an underlying disease or a complication or the occurrence of an unknown disease; (*3*) if any adverse event that is ≥ grade 3 of the Common Terminology Criteria for Adverse Events (CTCAE) ver. 4.0 occurs; (*4*) if the patient has been in the intensive care unit for more than 1 month; and (5) if the investigators judge that it is inappropriate for the patient to continue participating in the study for any other reason.

### Ethical considerations

This protocol was approved by the Clinical Research Review Board of Nagasaki University (CRB7180001), which is certified by Japan’s Ministry of Health, Labor, and Welfare (approval no. CRB19-0015). The study is registered in the Japan Registry of Clinical Trials as jRCTs071190051. The study will be conducted in accordance with the 7th revision of the Declaration of Helsinki 2013 and the Clinical Trials Act enforced in April 2020 in Japan. Any protocol modifications will be communicated to the Clinical Research Review Board at Nagasaki University and registered on the jRCT.

### Setting and study timeline

All participants will be recruited at Nagasaki University Hospital. Each participant’s information, as listed below, is collected at the prescreening for enrollment (visit 1): age, sex, height, body weight, history, comorbid complications, duration of cirrhosis, and history of cirrhosis treatment. Blood pressure and pulse rate will be measured in a sedentary position at every visit (visits 1–5, and, if withdrawing from the study, at withdrawal). As shown in Table 1, the participants will undergo examinations by blood tests, InBody 770 body composition analysis, and handgrip and quadriceps strength evaluations at enrollment (visit 1) and 1 month (visit 4) and 3 months (visit 5) thereafter. Similarly, they will undergo an examination of quadriceps strength at every visit, except at the start of BCAA (visit 2). When participants need to be withdrawn from the study for any reason, their vital signs will be checked, and they will undergo blood tests within 4 weeks after discontinuing the investigation. Blood specimens are to be obtained at visits 1, 4, and 5.

**Table 1:**
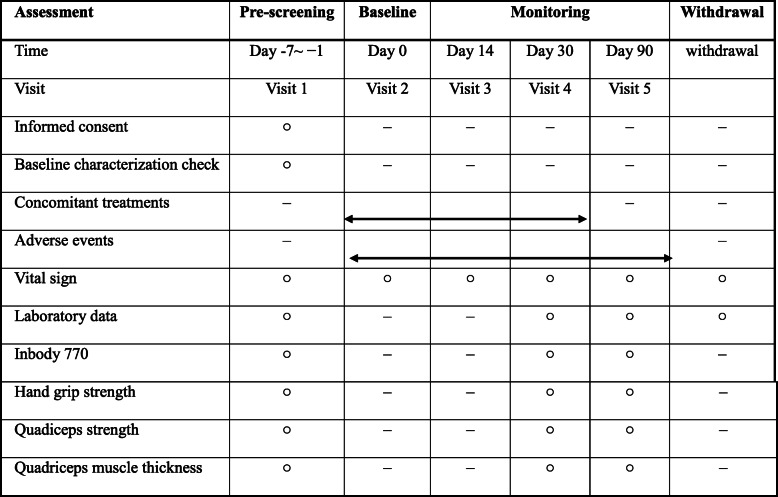
The schedule of pre-screening, interventions, and assessments

○ Will be done. A range of ±3 days is allowed for each visit dayThe examination at withdrawal is to be performed within 4 weeks from the date of withdrawal

### Laboratory measurements

At visits 1, 4, and 5, we will measure the patients’ complete blood count, prothrombin time-international normalized ratio, and plasma levels of aspartate aminotransferase, alanine aminotransferase, total bilirubin, albumin, C-reactive protein, hyaluronic acid, type IV collagen, *Wisteria floribunda* agglutinin-positive Mac-2-binding protein (WFA^+^-M2BP), ammonia, and cystatin C.

### Randomization and allocation concealment

A randomized, open-label, parallel pilot trial design has been set up. On the day on which the study drug is to be firstly administered (visit 2), i.e., after the eligibility assessment, each participant will be sequentially allocated to either the NMES and BCAA group or the control group (NMES only), in a 1:1 ratio, using blocked randomization stratified by age, sex, albumin level, and Child-Pugh score. The allocation will be conducted by the principal investigator using the Internet Data and Information Center for Medical Research (INDICE) system of UMIN.

### Interventions

#### NMES and BCAA group

Participants allocated to the NMES and BCAA group will undergo NMES within 7 days after LDLT and will be given a BCAA-enriched snack (Aminoleban EN, Otsuka Pharmaceutical Co., Tokyo, Japan; 100 mg/day) after leaving the intensive care unit (ICU) daily for 1 month.

#### Control group

Participants allocated to the control group will undergo only NMES within 7 days after LDLT for 1 month in addition to their previous treatment.

Both groups will receive muscle stimulation via placement of rectangular electrodes (90 × 50 mm) over the motor points of the quadriceps muscles (~ 5 cm distal to the inguinal fold and 15 cm proximal to the upper border of the patella) bilaterally. The stimulator (Pulsecure-Pro KR-7; OG Giken Co., Tokyo, Japan) delivers biphasic, symmetrical impulses (45 Hz; 400-μs pulse duration; 12 s on and 6 s off; intensity, 40–80 mA). The amplitude is increased to elicit visible muscle contractions and to the maximum level tolerated by the patients. Neuromuscular electrical stimulation sessions in both groups are conducted for 30 min per session, once per day, every weekday over a 4-week period, by a physical therapist, beginning on postoperative day 1 and continuing through postoperative day 30.

During this study, all intervention procedures will be checked by the attending physicians and a transplant coordinator in the Nagasaki University Hospital to improve adherence to intervention protocols.

### Adverse events

All adverse events (AEs) that occur during the trial will be recorded on a case report form and will be reviewed as part of central data monitoring. Investigators will explain AEs to the participants and offer appropriate care. All treatments will be performed within the Health and Medical Services during this study. If serious AEs, such as death, a life-threatening condition, hospitalization, sequelae, disability, and congenital illness, occur during the trial, the principal investigator will report the AE to the Minister of Health, Labor, and Welfare of Japan and the certified review board within 15 days.

### Data collection, data management, and monitoring

All data will be recorded in a case report form (CRF) by either the investigator or the clinical research coordinator. Only the patient identification number will be recorded in the CRF to ensure anonymization is maintained. The list associating the case number to personal information, to enable records to be identified, will be maintained in a secure fashion by the principal investigator. During the study, authorized investigators will make regular site visits to review protocol compliance, conduct source data verification, assess laboratory procedures, and ensure that the investigation is being conducted according to protocol requirements. Once the data are checked, they will be fixed by the trial steering committee (TSC). The TSC provides overall supervision for the trial to ensure that it is conducted in accordance with the rigorous standards established in the guidelines of the Clinical Trials Act. TSC comprises three researchers (MH, MF, and RS), who will meet and analyze the ongoing results of the research. No public organizations are involved in the study development.

### Statistical analyses

As stated in the Objectives section, the primary outcome of this study is the extent of the effects of BCAA on skeletal muscle mass after LDLT. Hence, the principal data analysis will be conducted using the set of participants with sufficient exposure to the allocated drugs. Therefore, we will use a per-protocol set (PPS). The same analysis will be conducted on the full analysis set (FAS). The respective analysis sets are defined as follows: the intention-to-treat (ITT) population is defined as all participants registered for this trial; the safety analysis set (SAS) population is defined as the participants in the ITT population that are administered NMES and BCAA-enriched snack at least once. The full analysis set (FAS) population is defined as the participants in the SAS for whom data about the predicted bone strength after the administration of NMES and BCAA-enriched snacks at one or more scheduled visits are available. PPS is a subset of FAS that excludes patients with major protocol violations and, thus, represents greater compliance with the protocol. Other criteria for the PPS will be determined before the database lock of this study. As a safety analysis, tabular summaries of AE incidence will be created. All hypothesis testing will be conducted at a significance level of 0.05 (two-sided). The data collected will be summarized using the arithmetic mean, the standard deviation, and the quantiles, and all statistical analyses are to be performed using JMP 14.0 software (SAS Institute Japan, Tokyo, Japan).

### Plans for communication of trial results and data access

Plans for communication of trial results include presentation at one or more national or international scientific meetings and publication in a peer-reviewed journal. The investigators plan to eventually make the final trial data set publicly available.

## Discussion

The primary purpose of this study is to determine whether NMES and BCAA combination therapy contribute to the recovery of skeletal muscle mass in patients with decompensated cirrhosis after LDLT.

Sarcopenia is one of the most common complications of several chronic diseases, including chronic liver disease. The recent definition of sarcopenia includes the presence of both low skeletal muscle mass and poor skeletal muscle function [18]. Sarcopenia has been associated with mortality in patients who have undergone LDLT; perioperative nutrition therapy has been shown to improve overall survival in patients with sarcopenia significantly [19]. In other words, increasing skeletal muscle mass and enhancing its function are essential points for promoting postoperative weaning after LDLT and improving overall survival.

BCAA are the essential amino acids valine, leucine, and isoleucine, which form the substrates for protein synthesis and energy generation in skeletal muscle [20, 21]. Several reports have shown that BCAA supplementation helps patients with liver cirrhosis recover from protein-energy malnutrition, raise serum albumin levels and subsequently improve the quality of life and survival [22, 23]. Previous reports have shown recovery of muscle mass after LDLT with the administration of BCAA [16]. One possible reason for the effect of BCAAs on sarcopenia is that leucine, one of the BCAAs, activates the rapamycin signaling pathway, which is involved in protein synthesis in muscle, and further stimulates pancreatic beta cells to release anabolic insulin in skeletal muscle [24].

Early administration of nutrition after transplant has been associated with a worse prognosis among seriously ill patients, suggesting that protein loading during the hypercatabolic state may negatively influence prognosis [25]. However, a previous study reported that the nutritional state and metabolism were improved by BCAA supplementation that was initiated on post-LDLT day 3 for a duration of 4 weeks [16]. BCAA supplementation did not improve the length of stay in the ICU or the total days of hospitalization. Based on this evidence, we will initiate BCAA supplementation after discharge from the ICU and not earlier in the course of post-LDLT recovery.

In recent years, NMES has been used to improve muscle strength and hypertrophy by contracting muscles with percutaneous low-frequency electrical stimulation. It has been reported that NMES improved muscle strength of the lower limb muscle group in seriously ill patients [26]. We have previously reported that NMES, combined with conventional physical therapy, significantly improved muscle mass in post-LDLT patients, as compared with physical therapy alone [15]. However, quadriceps muscle strength after LDLT was not sufficiently recovered, and ADL was not improved by this method. Therefore, we will investigate whether the addition of BCAA to NMES can improve quadriceps muscle strength and ADL post-LDLT.

There are some limitations to the trial. First, the sample size is small (*n* = 50). Second, the follow-up period of the examination may be insufficient to derive a definitive conclusion regarding the effect of NMES and BCAA therapy on sarcopenia after LDLT. Third, we cannot deny that other factors, such as postoperative complications, will influence the participants’ muscle atrophy.

Despite these limitations, our planned research also has some strengths. There has been no study assessing the effect of NMES and BCAA therapy on patients with decompensated cirrhosis. We will be able to elucidate the effect of NMES and BCAA treatment on sarcopenia using the InBody 770, which provides precise information on body composition. Additionally, ultrasonography and quadriceps dynamometer measurements will be useful for the evaluation of muscle atrophy.

In conclusion, LDLT is an effective treatment for decompensated liver cirrhosis patients showing poor improvement in response to medical treatment. However, this treatment is often accompanied by advanced invasion, which often results in a decline of ADL, such as a disuse syndrome, requiring long-term hospitalization. If the combination of NMES and BCAA is shown to be more effective than conventional treatment, it is expected to lead to better ADL and earlier discharge of patients after LDLT.

## Trial status

The recruitment is to be conducted from March 2020 to December 2023. The current version of this protocol is version 1.0; it was updated on February 18, 2020.

## Data Availability

Not applicable.

## Abbreviations

ADL: Activities of daily living
ALP: Alkaline phosphatase
ALT: Alkaline aminotransferase
AST: Aspartate aminotransferase
BCAA: Branched-chain amino acid
BUN: Blood urea nitrogen
CRP: C-reactive protein
γ-GTP: Gamma-glutamyl transpeptidase
LDH: Lactate dehydrogenase
LDLT: Living-donor liver transplantation
NMES: Neuromuscular electrical stimulation
PT: Prothrombin time
SMI: Skeletal Muscle Index
SMM: Skeletal muscle mass
VFA: Visceral fat area
